# Molecular characterization of *Dirofilaria* spp. circulating in Portugal

**DOI:** 10.1186/s13071-017-2180-y

**Published:** 2017-05-19

**Authors:** Cátia Ferreira, Ana Afonso, Manuela Calado, Isabel Maurício, Ana Margarida Alho, José Meireles, Luís Madeira de Carvalho, Silvana Belo

**Affiliations:** 1 Global Health and Tropical Medicine, GHTM, Instituto de Higiene e Medicina Tropical, IHMT, Universidade Nova de Lisboa, UNL, Lisboa, Portugal; 2 CIISA, Faculdade de Medicina Veterinária, Universidade de Lisboa (ULisboa), Lisboa, Portugal

**Keywords:** *Dirofilaria*, PCR, Internal transcribed spacer, Dog, Portugal

## Abstract

**Background:**

Dirofilariosis is a potentially zoonotic parasitic disease, mainly transmitted by mosquito vectors in many parts of the world. Data concerning the canine *Dirofilaria* species currently circulating in Portugal is scarce. Thereby, a large-scale study was conducted to determine the *Dirofilaria* spp. present in Portugal, based on a molecular approach, and also to optimize a reliable and highly sensitive species-specific polymerase chain reaction (PCR) assay that could be used for the simultaneous detection and differentiation of *Dirofilaria immitis*, *Dirofilaria repens*, and other concurrent filarial species in animal reservoirs.

**Methods:**

Blood samples were collected from three districts of Portugal (Coimbra, Santarém and Setúbal) between 2011 and 2013. Samples were tested using rapid immunomigration tests (Witness® *Dirofilaria*), modified Knott’s technique and acid phosphatase histochemical staining. In addition, molecular analysis was performed by amplification of the internal transcribed spacer (ITS) region using two different PCR protocols, specific for molecular screening of canine filarial species.

**Results:**

Of the 878 dogs sampled, 8.8% (*n* = 77) were positive for *D. immitis* circulating antigen and 13.1% (*n* = 115) positive for microfilariae by the modified Knott’s technique. Of the 134 samples tested by acid phosphatase histochemical staining, 100 (74.6%) were positive for *D. immitis*. Overall, 13.7% (*n* = 120) were positive by PCR for *D. immitis* by ITS2, of which 9.3% (67/720) were also positive by ITS1. ITS2 PCR was the most sensitive and specific method, capable of detecting mixed *D. immitis* and *A. reconditum* infections. Heterozygosity, in the form of double peaks, was detected by sequencing of both ITS regions. No *D. repens* was detected by any of the diagnostic methods.

**Conclusions:**

The present study confirmed *D. immitis* as the dominant species of the genus *Dirofilaria* infecting Portuguese dogs, based on sequencing of ITS1 and ITS2 PCR fragments. Additionally, ITS2 PCR was the most adequate method for diagnosis and prevalence estimation.

## Background

Dirofilariosis is a potentially zoonotic filarial parasitic disease, present in several parts of the world, transmitted mainly by mosquito vectors. The species *Dirofilaria immitis* and *Dirofilaria repens* (Filarioidea, Onchocercidae) are widely present in the Mediterranean basin and are the causative agents of cardiopulmonary and subcutaneous dirofilariosis, respectively. Both nematodes are transmitted by mosquito species of the family Culicidae and can infect domestic and wild canids and felids, causing severe pathological effects [1]. *Dirofilaria immitis* is considered the most virulent filarial species in dogs, as the long-lived adult worms reside in the right ventricle and pulmonary artery, leading to pulmonary hypertension, congestive heart failure and even death [2, 3]. Instead, *D. repens* adult forms live in subcutaneous tissue, where they cause dermatological problems, such as multifocal nodular and prurigo papularis dermatitis. Moreover, both species may also infect humans. *Dirofilaria immitis* pre-adult forms can cause pulmonary nodules and *D. repens* adult/pre-adult stages may induce subcutaneous and ocular lesions [4, 5]. Other less known canine filarial parasites, such as *Acanthocheilonema dracunculoides* (tick- and fly-transmitted) and *Acanthocheilonema reconditum* (flea- and lice- transmitted), may also infect companion animals [6, 7]. Adult *A. reconditum* and *A. dracunculoides* reside in the peritoneal cavity and adipose tissue of the host, and thus seem to be less virulent for canine reservoirs. Nevertheless, *A. reconditum* has also been reported in humans [8].

These filarial species release circulating microfilariae (Mf) in the blood of their definitive hosts. These Mf can be diagnosed by microscopy through specific morphological identification or Mf histochemical staining [9, 10]. Other diagnostic methods are also available, such as detection of circulating adult female antigens (currently only for *D. immitis*) and molecular approaches [1, 11, 12]. Modified Knott’s and acid phosphatase histochemical staining tests of blood smears remain the most commonly used parasitological tests for Mf detection, but are labour-intensive and require expertise. Thus, the prevalence of *Dirofilaria* spp. can be over-estimated if other filarial species are present and misidentified [13, 14]. Molecular protocols have been developed for reliable detection and differentiation of filarial species, in particular, a species-specific PCR assay and multiplex PCR and restriction fragment length polymorphism (RFLP) assays for simultaneous detection of different *Dirofilaria* spp., either in the vector or in blood [12, 14–21].

Canine dirofilariosis due to *D. immitis* is known to be endemic and widely distributed in Portugal, with prevalence ranging between 0.9 and 27.3% in mainland regions to over 30% in Madeira Island [22–25]. *Dirofilaria repens* was recently detected for the first time, in a dog, presenting as mixed infection with *D. immitis* [26]. This is a worrying finding, as the occurrence of autochthonous infections in domestic animals and the numbers of notified human cases of dirofilariosis, mainly attributed to *D. repens*, have increased substantially in several European countries in recent years [5, 27, 28].

The aim of the present study was to identify the *Dirofilaria* species currently circulating in Portuguese dogs through an optimised reliable and highly sensitive species-specific PCR assay for the simultaneous detection and differentiation of *D. immitis*, *D. repens* and other concurrent filariids in animal reservoirs.

## Methods

### Study areas and canine sampling examination

The study areas, as well as the clinical and parasitological procedures, were as previously described [25]. Briefly, canine surveys were conducted in kennels (run by local authorities or animal protection associations) in three districts of Portugal: Coimbra (northern-Centre region), Santarém (central-Centre region) and Setúbal (southern-Centre region) during three consecutive years: 2011, 2012 and 2013. Three surveys were carried out each year, in spring (March-April), summer (July-August) and autumn (October-November). Only dogs older than 6 months of age and residing in the kennels for at least 6 months were included.

### Direct and serological tests

For clinical and parasitological examination, dogs were randomly sampled in each kennel. Physical examination was performed prior to blood collection. Blood was collected from the cephalic vein (5 ml) and stored (2.5 ml) with either anticoagulant EDTA or in a dry tube, and later processed for parasitological, serological and molecular analyses. The modified Knott’s technique (KN) and the acid phosphatase histochemical staining test (AP) were used for microscopic detection and identification of Mf in blood smears. The commercial kit WITNESS® *Dirofilaria* (WT) (Synbiotics, San Diego, CA, USA) was employed for detection of *D. immitis* circulating antigen in serum.

### Molecular analysis

#### DNA isolation

DNA was extracted from whole blood using CTAB (cetyltrimethyl ammonium bromide) method, adapted from Stothard et al. [29]. Briefly, 100 μl blood with EDTA (ethylenediamine tetraacetic acid) was incubated with 600 μl CTAB buffer and 0.2 mg proteinase K (Bioline, London, UK) at 56 °C for 2 h, with agitation. DNA precipitation was done with 0.6 ml absolute ethanol and the pellet hydrated in 50 μl TE buffer (10 mM Tris, 1 mM EDTA, pH 7.0). DNA samples were stored at -20 °C until further use.

For *D. immitis* positive control, DNA was extracted, as above, from a small macerated section of two adult worms. For *D. repens* positive control, DNA was extracted from infected canine blood and from a worm (kindly provided, respectively, by Prof. Eva Fok, University of Veterinary Medicine, Budapest, Hungary, and by Prof. Claudio Genchi, University of Milan, Italy). Deionised water was used as a PCR negative control.

#### DNA amplification

The ribosomal internal transcribed spacer (ITS) region was amplified using two different PCR protocols for molecular screening of canine filarial species. The internal transcribed spacer 1 (ITS1) region was amplified using a semi-nested PCR as described by Nuchprayoon et al. [30]. Briefly, primers FL1-F and FL2-R were used in a first-round PCR to amplify the entire ITS region, and primers FL1-F and Di5.8S 660-R in a second-round PCR to amplify the ITS1 region, with expected amplification fragment sizes for *D. immitis*, *D. repens* and *A. reconditum* of 595, 602 and 446 bp, respectively. Amplification of the internal transcribed spacer 2 (ITS2) region was carried out using the primers DIDR-F1 and DIDR-R1 [21], with expected amplification product sizes of 542, 484, 578 and 584 bp for *D. immitis*, *D. repens*, *A. reconditum* and *A. dracunculoides,* respectively. All PCR reactions were performed in 25 μl reaction mixtures, containing PCR buffer (Promega, Madison, WI, USA), 6 mM MgCl_2_ (Promega), 10 pmol of each primer, 12 mM dNTPs (Promega), 2.5 U GoTaq® DNA polymerase (Promega), 10–40 ng of template DNA in deionized water. The temperature profile for both steps of the semi-nested ITS1 PCR was: 94 °C for 5 min, followed by 35 cycles of 94 °C for 30 s, 58 °C for 30 s and 72 °C for 45 s, with a final extension step at 72 °C for 10 min. Amplification of the ITS2 region had the following temperature profile: 94 °C for 2 min and 32 cycles of 30 s at 94 °C, 30 s at 60 °C and 30 s at 72 °C, with a final extension step for 7 min at 72 °C. Amplification products were separated by electrophoresis in 2% agarose gels, stained with ethidium bromide, and visualized under UV light.

PCR analytical sensitivity was tested with serial dilutions (by a factor of 10) of DNA from a female adult worm of *D. immitis*, canine blood infected with *D. repens* and from dog blood samples with positive PCR (ITS1/ITS2) for *D. immitis*.

#### DNA sequencing and phylogenetic analysis

PCR amplicons were purified using a commercial kit (Qiagen, QIAquick PCR Purification Kit, Germantown, USA) and sequenced commercially (Macrogen, Seoul, South Korea) using the PCR primers. A BLAST search was performed to confirm species identity of the sequenced amplicons. Homologous sequences available in GenBank/EMBL/DDBJ databases were retrieved by BLAST and all sequences were aligned in BioEdit 7.2.5 [31]. Some sequences exhibited regions of double peaks, and haplotypes were inferred manually to correspond to homozygous sequences in circulation (for ITS1), or using the programme PHASE [32] with 100 iterations, 100 thinning interval and 100 ‘burn-in’ settings (for ITS2).

Phylogenetic relationships were estimated using MEGA 7.0 [33], based on an alignment of regions with no gaps. The phylogenetic trees were inferred by the Maximum Parsimony method parameter, CNI (level = 1) with initial tree by random addition (10 reps) with 1,000 bootstrap replicates and a cut-off value of 74%.

### Statistical analysis

Pearson’s Chi-square and Fisher’s exact tests were used to evaluate the differences between the proportions of species-specific infected dogs detected by each PCR protocol, among different age groups (0.5–3 years, > 3–6 years, > 6 years), gender and district as compared with parasitological and serological tests. Level of agreement was calculated using Cohen’s kappa coefficient (*K*). Statistical analysis was carried out using statistical software SPSS 15.0 for Windows 10.0; a *P* < 0.05 was considered significant.

## Results

Overall, 878 dogs (400 males and 478 females) were sampled from the three areas, Coimbra (*n* = 268), Santarém (*n* = 465) and Setúbal (*n* = 155). The dogs were 0.5 to 16 years old, with a median age of 4.5 years (IQR 2.5–7.0).

The analytical sensitivity of ITS1-PCR and ITS2-PCR were, respectively, 4.5 and 0.09 pg DNA from female adult *D. immitis*, 118 and 200 pg DNA from *D. repens*-infected dog blood, and 250 and 2.5 pg DNA from a *D. immitis*-infected dog. Statistical sensitivity (i.e. the proportion of true positives) and specificity (i.e. the proportion of true negatives) of ITS2-PCR were significantly higher (McNemar test, *P* < 0.05) than of ITS1-PCR.

In the 720 dogs tested using both PCR targets, samples positive for ITS1-PCR were also positive for ITS2-PCR. Higher analytical sensitivity was observed for ITS2-PCR, with 12.9% of the blood samples positive for *D. immitis* (Table 1). Using ITS2-PCR it was possible to amplify, not only both species of *Dirofilaria* spp., but also *A. reconditum* in canine blood. Two samples that were ITS1-PCR-positive for *D. immitis*, were characterized by ITS2-PCR-RFLP as *A. reconditum* (accession number ENA: HG964682–HG964684) and were not included in the calculations. DNA from species (*D. immitis* and *Acanthocheilonema* spp.) was detected by ITS2-PCR in two samples.

**Table 1:** Performance of ITS1 *vs* ITS2-PCR in 720 dog samples

	*D. immitis*		*A. reconditum*	Mixed	*K*	*P*
	Positive (%)	Negative (%)	Positive (%)	Positive (%)		
ITS1	67 (9.3)	652 (90.6)	1 (0.1)	0 (0)	0.767	0.037
ITS2	93 (12.9)	620 (86.1)	5 (0.7)	2 (0.3)		

*K*: level of agreement (*K* = 0.767, *P* = 0.037) between each pair of tests (positive or negative results in both tests)

The performance of the PCR with the highest analytical sensitivity (ITS2) was compared with serological and direct parasitological tests for all samples (Table 2). Out of the 878 samples tested, *D. immitis* circulating antigen was detected in 77 (8.8%) by WT, whereas Mf were found in 115 (13.1%) stained slides by KN method. Samples with inconsistent results between WT and KN (*n* = 19 WT-positive, KN-negative) and KN-positive blood slides (*n* = 115) were submitted to AP analysis (*n* = 134). Out of the 134 stained slides, *D. immitis* Mf were identified in 100 (74.6%) and *A. reconditum* in two (1.5%). *Dirofilaria repens* was not identified in blood smears through any method.

**Table 2:** Prevalence of filarial infection according to the diagnostic assays performed

	Total no. of samples	*D. immitis*	*Acanthocheilonema* spp.	Mixed
		Positive (%)	Positive (%)	Positive (%)
Witness	878	77 (8.8)	–	–
Knott	878	115 (13.1)	–	–
Acid phosphatase	134	100 (74.6)	2 (1.5)	–
ITS2	878	120 (13.7)	5 (0.6)	2 (0.2)

ITS2-PCR and KN presented the highest level of agreement (Cohen’s kappa coefficient), which was lower, but also statistically significant, between ITS2-PCR and WT (Table 3).

**Table 3:** Agreement between ITS2-PCR in relation to direct and serological methods

Test	Total no. of samples	Positive (%)	Negative (%)	*K*	*P*
Witness	878	65 (84.4)	739 (92.3)	0.593	0.042
Knott	878	107 (93.0)	750 (98.3)	0.930	0.018*
Acid phosphatase	134	97 (97.0)	14 (43.8)	0.513	0.088

*K*: level of agreement between each pair of tests (positive or negative results in both tests)

*P < 0.05

### Characterization of *Dirofilaria* spp.

**Fig. 1:**
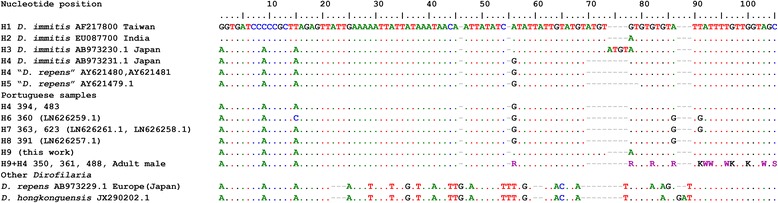
Alignment of heterozygous ITS1 sequences of *D. immitis* from Portuguese canine samples. The haplotypes were inferred based on circulating haplotypes, considering the most parsimonious hypothesis that at least one haplotype is the same as the most common in circulation in the population. The first position on the alignment corresponds to position 604 of the first sequence, AF217800, reversed. The nucleotide codes K, R, S, and W, correspond, respectively to T/G, A/G, G/C and A/T

Sequences obtained from selected ITS1 and ITS2 PCR products were analysed and deposited in GenBank under accession numbers LN626257–LN626259 and LN626261 (samples 391, 623, 360 and 363, respectively; complete ITS region); KY014643–KY014648 (samples 483, 394, 350, 361, 488, female adult worm, respectively; ITS1) and KY644132–KY644141 (samples 1, 7, 8, 29, 52, 483, 723, 732, 758, 846, respectively; ITS2). Three out of nine ITS1 sequences analysed from PCR products obtained from canine blood were found to have a string of double peaks, as did a female *D. immitis* worm (Fig. 1). The haplotypes for the ITS1 heterozygous sequences were inferred manually by assuming one sequence to be identical to the most common homozygous sequence (or haplotype) found in local samples, which in this case was H4 (Fig. 1). H4 was found in one sample from Japan, as well as the sequences AY621480.1 and AY621481.1, labelled as *D. repens* in the GenBank database (Fig. 1). The other inferred haplotype (H9) presented similarities with sequence EU087700, from India, but only from position 50 onwards in the alignment in Fig. 1. The region up to position 20 was more similar to other Portuguese and Japanese samples.

**Fig. 2:**
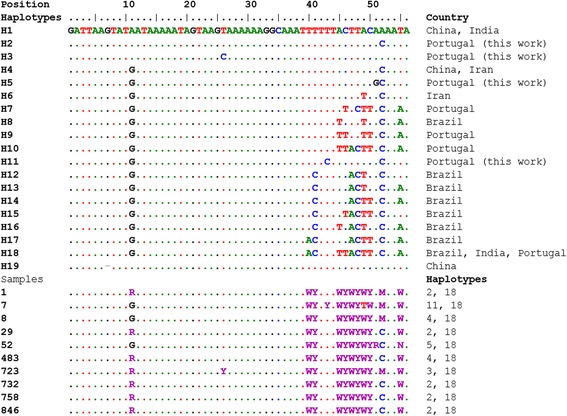
Alignment of heterozygous ITS2 sequences of *D. immitis* from Portuguese canine samples. Haplotypes 7, 9, 10 and 18 include Portuguese sequences with codes LN626262-7 that are from infected mosquitoes. Haplotypes were inferred using the programme PHASE, by comparison with homozygous sequences, with 100 iterations, 100 thinning interval and 100 burn-in settings. The first position on the alignment corresponds to position 162 of the sequence, EU087699 (H18). The nucleotide codes are as standard (M, R, Y and W, correspond, respectively to A/C, A/G, C/T and A/T). Base assignment in position 11 (R) is considered uncertain (probability of 58-9%), as is for position 26 (Y) of sample 723 (50%), position 43 (Y) of sample 7, and position 51 (R) of sample 52 (50%). Haplotypes: H1: JX481279, JX866681, EU182329; H4: U182331, JN084166, JX889634, JX8896351, JX889636, JX889637, JX889638; H6: JN084168; H7: LN626265; H8: FJ263455; H9: LN626264/66; H10: LN626262; H12: FJ263458/66/67; H13: FJ263459/60/63; H14: FJ263457/64; H15: FJ263468, H16. FJ263461; H17: FJ263465; H18: EU087699, FJ263456, LN626263.1, LN626267.1; H19: EU182330. Haplotypes H2, H3, H5 and H11 result from inference

ITS2 sequences also presented several heterozygous sites, in particular after an A and T rich region. By comparison with sequences obtained from a BLAST search, the ITS2 sequences obtained here were most similar to *D. immitis* and quite distinct from *D. repens* sequences present in GenBank, as revealed by phylogenetic analysis (not shown). Statistical reconstruction of haplotypes by comparison with sequences present in GenBank identified 19 different haplotypes (Fig. 2). All heterozygous Portuguese samples included haplotype H18, which was present in sequences from India and Brazil (dog). Five samples had H2 as the other haplotype, which has no correspondence in the database, and two samples had haplotype H4, which was present in sequences from China (red panda) and Iran (dogs). The main difference between haplotype H18 and other haplotypes was a gap of two nucleotides in a T repeat. The other three haplotypes identified (H3, H5 and H11) were not found elsewhere in the database.

A BLAST analysis of the entire ITS region showed greatest similarities to *D. immitis*, with a sequence similarity that ranges from 89% to 97% with sequences available at NCBI database (JX866681.1; DQO18785.1; JX866681.1; FJ263464.1; FJ2634571; HM126606.1).

### Pattern of canine *D. immitis* infection related to gender and age

Based on ITS2-PCR, the prevalence of *D. immitis* infection found in males (63/400; 15.8%) was significantly higher (*P* = 0.032) than in females (57/478; 11.9%). There were also significant differences (*P* = 0.01) in prevalence between age groups; the highest was found in dogs > 6 years of age (76/426; 17.8%), followed by the group with > 3–6 years of age (32/265; 12.1%) and the lowest in the 0.5–3 years age group (12/187; 6.4%). Similarly, statistically significant differences (*P* = 0.016) in prevalence were found between districts: Setúbal had the highest (29/155; 18.7%), followed by Santarém (63/455; 13.8%) and Coimbra (28/268; 10.4%).

## Discussion

The application of molecular analyses targeting filarial genomic DNA in blood samples proved in this work to be a highly sensitive and specific analytical tool for the diagnosis and simultaneous characterization of canine filarial infections [19, 21, 34]. In comparison with serological and parasitological methods, PCR provided more reliable data for clinical and epidemiological purposes.

In the present study, the ITS2-PCR had higher analytical sensitivity and specificity than the ITS1-PCR, particularly in samples with low microfilaremia (< 5 Mf per 20 μl of blood), for which ITS1 amplification failed or gave non-specific results. In addition, even in single or mixed infection cases, species identification of the filariae in infected dogs was also more consistent for ITS2 (Table 1).

Although parasitological and serological methods are still the most frequently used techniques for the diagnosis of canine dirofilariosis [35], the present results showed that ITS2-PCR performs better in different aspects (sensitivity, specificity and species identification), thus contributing to improve diagnosis and to provide a more accurate estimation of the epidemiological pattern in the country. The ITS2-PCR assay detected mostly *D. immitis* single infections, but also 5 (0.6%) cases of *A. reconditum* and 2 (0.2%) of mixed infections (*D. immitis* + *A. reconditum*) (Table 2). ITS2-PCR was the most sensitive method, but with very similar analytical sensitivity to KN, followed by WT.

Agreement was strongest and statistically significant between PCR-ITS2 and KN test, but the molecular assay has the advantage of detecting filarial DNA in co-infected animals. Agreement between ITS2-PCR and AP or WT was much weaker. Serology is still useful for epidemiological surveys, as it can be faster and easier to use, allowing results launching to dog owners in a short time. However, detection of *D. immitis* DNA in unapparent infections can complement serology in canine surveys.

Molecular results based on ITS2-PCR also confirmed previous findings of *D. immitis* infection in dogs related to sex, age, regional distribution and prevalence [25]. In fact, previous results based on WT, KN and AP tests have also shown a higher prevalence in male dogs, older than 6 years of age and from Setúbal, confirming the North-South prevalence increase trend, as reported previously based on a fast serological diagnostic kit [24].

Sequence analyses of ITS1 and ITS2 fragments identified a high number of samples with at least two different alleles, which differed in sequence length, as per the inferred haplotype sequences. Although at least one of the alleles detected in each ITS region had also been found in isolates from Portugal and other regions, some samples had inferred haploid sequences that were described here for the first time. It was not possible to determine if the parasites were heterozygous or if these were cases of mixed infections in the dog. However, one adult worm presented the same heterozygous profile for ITS2, and the same ITS heterozygous patterns had been observed in the PCR product from a mosquito in Portugal, *Aedes detritus* (*s.l*.) [36]. PCR on individually isolated Mf should clarify this issue. It is of note that some ITS1 sequences in the database had been erroneously labelled as *D. repens*, when, in fact, they correspond to *D. immitis*. Such observations raise the question over earlier publications of *D. repens* occurrence or prevalence based on this target.

*Acanthocheilonema* spp. are also common filarial nematodes that infect dogs in Europe and, although less virulent for animals, identification of Mf of this species in blood samples by microscopy is complex and misdiagnosis as *D. immitis* can often occur. The species-specific ITS2-PCR applied in this study detected a 0.8% prevalence of *A. reconditum*, which is similar to the prevalence found by Menn et al. [37].

The present study showed that *D. immitis* remains, so far, the dominant species of *Dirofilaria* genus in Portugal, as confirmed by sequencing of ITS1 and ITS2 fragments from canine blood samples. These results are consistent with the results by Ferreira et al. [36], who only detected *D. immitis* in mosquito vectors collected in the same time period in the same districts.

However, *D. repens* has recently been identified in one dog in the Algarve [26] in southern Portugal. The Algarve has the highest number of days per year with suitable conditions for *Dirofilaria* transmission [38] and it is, thus, likely that it has been the point of introduction of this species in Portugal. Although with very low prevalence, the presence of *D. repens* in the Algarve is worrying since this species has been implicated in the increasing number of reports of human dirofilariosis in Europe [28]. Such introduction was expected, as is the establishment and an increase in prevalence of this parasite species in Portugal, given the ongoing north- and eastward expansion of both *Dirofilaria* species that has been observed. Such expansion has been mainly attributed to global warming, as well as environmental changes, which promote the expansion of mosquito vectors, along with the increased international mobility of infected vertebrates [27, 39–41]. Moreover, many wild animals can also act as sylvatic reservoirs for *Dirofilaria* spp., thus maintaining transmission of this parasite. In Portugal, the prevalence of *D. immitis* in red foxes, as determined by necropsy, has ranged from 3.2% in northern-Centre locations, such as Coimbra [42], to 11.8% in southern and central-Centre districts, such as Santarém and Setúbal [43]. Additionally, in a national serological survey conducted in red foxes, 8.5% were positive for *D. immitis* circulating antigen, with positive animals found in northern and southern areas of Portugal [44]. *Dirofilaria immitis* has also been reported in three Eurasian otters, *Lutra lutra*, in Portuguese natural freshwater habitats [45, 46] and, recently, in a collection of pinnipeds from Algarve [47].

## Conclusions

In conclusion, our data strongly suggest that *D. immitis* is the main etiological agent of dirofilariosis in Portugal and that PCR of the region ITS2, as applied here, could be a valuable tool for the diagnosis and screening of filarial infections in dogs, given its fast, accurate, specific detection and differentiation of *Dirofilaria* spp. from other concurrent blood microfilariae.

## Abbreviations

AP: Acid phosphatase histochemical staining test
CTAB: Cetyltrimethyl ammonium bromide
EDTA: Ethylenediamine tetraacetic acid
ITS: Internal transcribed spacer
KN: Modified Knott’s technique
Mf: Microfilariae
PCR: Polymerase chain reaction
RFLP: Restriction fragment length polymorphism
WT: Kit WITNESS® Dirofilaria
